# Dynamic network data envelopment analysis for university hospitals evaluation

**DOI:** 10.1590/S1518-8787.2016050006022

**Published:** 2016-04-27

**Authors:** Maria Stella de Castro Lobo, Henrique de Castro Rodrigues, Edgard Caires Gazzola André, Jônatas Almeida de Azeredo, Marcos Pereira Estellita Lins

**Affiliations:** I Serviço de Epidemiologia e Avaliação. Hospital Universitário Clementino Fraga Filho. Universidade Federal do Rio de Janeiro. Rio de Janeiro, RJ, Brasil; II Programa de Residência em Saúde Coletiva. Instituto de Estudos em Saúde Coletiva. Universidade Federal do Rio de Janeiro. Rio de Janeiro, RJ, Brasil; III Departamento de Engenharia de Produção. Instituto Alberto Luiz Coimbra de Pós-Graduação e Pesquisa de Engenharia. Universidade Federal do Rio de Janeiro. Rio de Janeiro, RJ, Brasil

**Keywords:** Hospitals, University, organization & administration, Efficiency, Organizational, economics, Programming, Linear, utilization, Health Services Evaluation

## Abstract

**OBJECTIVE:**

To develop an assessment tool to evaluate the efficiency of federal university general hospitals.

**METHODS:**

Data envelopment analysis, a linear programming technique, creates a best practice frontier by comparing observed production given the amount of resources used. The model is output-oriented and considers variable returns to scale. Network data envelopment analysis considers link variables belonging to more than one dimension (in the model, medical residents, adjusted admissions, and research projects). Dynamic network data envelopment analysis uses carry-over variables (in the model, financing budget) to analyze frontier shift in subsequent years. Data were gathered from the information system of the Brazilian Ministry of Education (MEC), 2010-2013.

**RESULTS:**

The mean scores for health care, teaching and research over the period were 58.0%, 86.0%, and 61.0%, respectively. In 2012, the best performance year, for all units to reach the frontier it would be necessary to have a mean increase of 65.0% in outpatient visits; 34.0% in admissions; 12.0% in undergraduate students; 13.0% in multi-professional residents; 48.0% in graduate students; 7.0% in research projects; besides a decrease of 9.0% in medical residents. In the same year, an increase of 0.9% in financing budget would be necessary to improve the care output frontier. In the dynamic evaluation, there was progress in teaching efficiency, oscillation in medical care and no variation in research.

**CONCLUSIONS:**

The proposed model generates public health planning and programming parameters by estimating efficiency scores and making projections to reach the best practice frontier.

## RESUMO

**OBJETIVO:**

Desenvolver ferramenta de avaliação de eficiência de hospitais universitários federais de perfil geral.

**MÉTODOS:**

A análise envoltória de dados, técnica de programação linear, constrói uma fronteira de melhores práticas pela comparação da produção observada dadas as quantidades de recursos despendidas. O modelo é orientado a produto, e considera retornos variáveis de escala. A análise envoltória de dados em redes considera variáveis de ligação que pertencem a mais de uma dimensão (no modelo, médicos residentes, internações ajustadas e projetos de pesquisa). A análise envoltória de dados dinâmica usa variáveis de transporte (no modelo, receita) para analisar o deslocamento da fronteira em anos subsequentes. Os dados foram coletados do sistema de informações do MEC, 2010 a 2013.

**RESULTADOS:**

Os escores médios de assistência, ensino e pesquisa no período foram: 58,0%, 86,0% e 61,0%, respectivamente. Em 2012, ano de melhor desempenho, para que todas as unidades atingissem a fronteira, seria necessário aumento médio de consultas de 65,0%; de internações, de 34,0%; de alunado de graduação, de 12,0%, de residência multiprofissional, de 13,0%, de pós-graduação, de 48,0%; de projetos de pesquisa, de 7,0%; além de queda de 9,0% de residentes médicos. No mesmo ano, para melhora da fronteira de produção assistencial, seria necessária a injeção de um aporte adicional de receita de 0,9%. Observou-se progressão da eficiência no ensino; oscilação na assistência e estagnação na pesquisa na avaliação dinâmica.

**CONCLUSÕES:**

O modelo proposto gera parâmetros de planejamento e programação em saúde pública por meio do cálculo dos escores de eficiência e das projeções necessárias para alcance das fronteiras de melhores práticas.

## INTRODUCTION

Hospitals are complex, multi-professional and interdisciplinary institutions, with specific technological density, according to the Brazilian National Policy for Hospital Care, implemented in the Health Care Networks. They are responsible for providing health care to users on an inpatient basis and via services covering health promotion, disease prevention, diagnosis, therapy, and rehabilitation^a^.

Barata et al.^1^ consider health to be a social right. They stress the complementary contribution of teaching hospitals in training health professionals to adequately tackle priority problems of the Brazilian population and developing research to help cope with these problems. Teaching hospitals include all hospital units, general or specialized, public or private, independent or attached to a higher education institution, which provide curricular activities in the field of health^b^. University Hospitals (UH) belong to a federal or state higher education institution, with coordinated activities in care, teaching, and research.

Since the 1990s, the funding and management crisis of Brazilian hospitals, in particular university hospitals, has been placed on the agenda, with important repercussions in care, teaching, and research activities. Initiatives by the Brazilian Federal Government to face the crisis were based on financial, material and human resources support, and the introduction of improved management practices, among them the National Policy for the Certification of Teaching Hospitals, the National Program for Restructuring Federal University Hospitals, and the creation of the Brazilian Hospital Services Corporation^b^ ^,^ ^c^ ^,^ ^d^. Such initiatives aimed to attain excellence in teaching hospitals in care, teaching, and research by establishing management goals that would ensure the best use of resources in carrying out their tasks.

The development of performance studies using efficiency frontiers is appropriate to analyze the use of resources in achieving objectives. Among efficiency studies applied to the field of health, data envelopment analysis (DEA) is the most frequently used technique (48.0% of publications)^5^.

This study aimed to develop an assessment tool to evaluate efficiency in federal general university hospitals. This tool considers coordination between teaching, care, and research and the monitoring of changes over time.

## METHODS

The model is based on dynamic network DEA to help decision-making processes among managers. A longitudinal analysis of interrelations is carried out between the dimensions of care, teaching, and research regarding their efficiency.

DEA is a linear programming technique first described by Charnes et al.^2^ (1978). It measures efficiency by comparing similar units (DMU or Decision Making Units) that use the same resources (inputs) and generate the same products (outputs). A DMU is considered efficient when, compared to the others, it produces more outputs with a fixed amount of inputs (output-oriented model), or uses fewer resources to generate a fixed amount of outputs (input-oriented model). Efficient DMUs receive the maximum score of 100% and shape the best practice frontier, which involves the set of compared units. A DMU is only considered efficient when it reaches the Pareto-Koopmans optimality frontier, in which it is not possible to increase an output (or reduce an input) without reducing another output (or increasing another input) at the same time.

In classic models, the frontier may consider constant returns to scale (CRS, when an increase in outputs proportional to the increase in inputs is expected) or variable returns to scale (VRS, usually preferred when comparing units very different in size and outcome scale), a format established *a priori* by the researcher. Being a deterministic technique, any distance from the frontier (score lower than 100%) results from inefficiency, i.e., from the difference between current (X%) and projected values (100-X%). The projection of inefficient DMUs considers the shortest distance from or the nearest point to the frontier, where similar efficient units are located as benchmarks^14^.

In the development of classic models, when the production process generates intermediate outputs that serve as inputs for new processes, network DEA can be used. Network DEA is composed of a family of DEA models that establishes linear constraints for each one of the analyzed dimensions. It is thus possible to consider link variables belonging to more than one dimension and generate efficiency scores for all of them^3^.

The models applied to case studies in the literature do not usually pay attention to projections in Pareto-inefficient regions of the frontier, and the projections with slacks in relation to the Pareto-efficient target result in efficiency estimation mistakes^8^. Prominent among the non-radial models that determine efficiencies based on Pareto-efficient targets are the SBM (Slacks-based Model) and Russel models. In this work, we chose to use the DEA model in SBM networks to evaluate efficiency, as proposed by Tone and Tsutsui^15^ ^,^ ^16^.

For longitudinal monitoring, dynamic DEA enables the generation of efficiency scores for distinct and consecutive times by considering efficiency at each moment and the frontier shift over time. In each studied period, the DMUs use up the same inputs and generate the same outputs. However, some outputs of a period (capital and human resources, for example) can serve as inputs for the following period. These link variables between periods are called carry-over, which can be treated as fixed or mutable according to frontier projection.

The network DEA and dynamic DEA models are integrated (DNSBM *–* Dynamic Network Slack-based Model) in this study. Vertically, it deals with multiple dimensions linked in a networks structure within each period, and, horizontally, each structure is combined in networks by carry-over variables that link two distinct periods. The model enables the evaluation of: a) overall and single-dimension efficiency, at each evaluated moment; b) changes in overall and single-dimension efficiency, over the studied period. A modified Malmquist Index was developed, which evaluates changes in individuals scores (catch-up component) and the technological frontier shift, or of all hospitals over time (frontier shift)^16^. Values higher than 1.0 mean performance progression in the interpretation of the index and its components; lower values mean frontier regression.

The DNSBM model used is output-oriented (aiming at increasing output) and considers variable returns to scale (given the different sizes of the analyzed hospitals). The network structure considers the relation between the dimensions of care, teaching and research, and the dynamic assessment analyzes the 2010-2013 period. The DMUs are the 31 federal general university hospitals, run by the Brazilian Ministry of Education (MEC), identified here by their respective universities. The variables were taken from the Ministry of Education Information System (SISREHUF).

**Figure 1:**
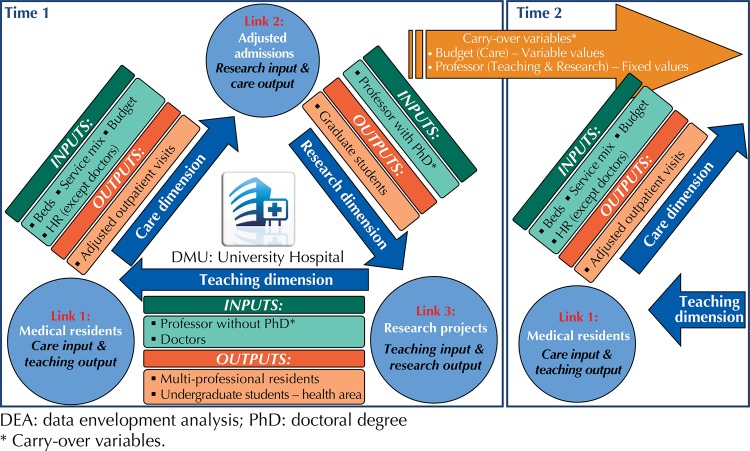
Diagram of the efficiency and variables study of the dynamics network DEA model. Brazil, 2010-2013.

Figure 1 illustrates the studied model schematically and Table 1 describes in detail the variables in the several dimensions: inputs, outputs, link and carry-over. No variables – other than link variables – were repeated in different dimensions.

**Table 1:** Study variables and dimensions. Brazil, 2010-2013.

Dimension	Variable	Observation	Type
Care	Number of beds	Total number of active beds per year.	Input
	Technology sum index, or Service mix	Sum of equipment and technology reported to the National Registry of Health Institutions. The first two variables, beds and service mix, can be jointly treated as capital proxyl^12^.	Input
	Human Resources (total, except doctors)	Number of staff, except doctors. Considers total workforce, excluding doctors. This variable highly correlates with the number of doctors, which will be considered in another dimension of the model.	Input
	Outpatient visits adjusted by complexity	Number of adjusted annual outpatient visits. A Complexity Index was created for adjustment, based on the percentage of high complexity procedures performed at outpatient level, then multiplied by the volume of visits.	Output
	Output budget (goals agreement with manager)	The annual output budget (in millions of reais) is the input defrayal for one year and the proxy output measure for the following year. Since negotiation of budget values is based on the historical output series of previous years, it is useful to program a more efficient funding system.	Carry-over
Teaching	Doctors	Number of doctors involved in service training activities in wards and outpatient departments.	Input
	Number of multi-professional residents	Total number of multi-professional residents only.	Output
	Sum of undergraduate students in all health areas	Total number of undergraduate students in all health professions in activity at the hospital. This output aims to emphasize the view of multidisciplinary activities.	Output
	Professor without doctoral degree	Number of professors with a masters, specialization or bachelor degree, mainly dedicated to teaching. Fixed variable, given the low variability from one year to another and the low control of the hospital management over the number of places.	Carry-over
Research	Number of students in graduate courses	Total number of enrolled graduate students.	Output
	Professor with doctoral degree	Number of professor with a doctoral degree. Fixed variable, given the low variability from one year to another and the low control of the hospital management over the number of places. Following the logic of not repeating a variable in more than one dimension, PhD professor is considered to be mostly dedicated to research.	Carry-over
Care - Teaching	Number of medical residents	Care input and teaching output. These doctors play an important role in the care workforce, and, at the same time, are new graduates undergoing service training.	Link
Care - Research	Admissions adjusted by complexity	Care output and research input. Annual admissions are the main care output and, at the same time, the basis for the development of clinical research and care technologies in the hospital. Complexity adjustment considers the percentage of high complexity procedures performed in the admissions unit.	Link
Research - Teaching	Research projects	Teaching input and research output. Annual number of projects approved by CEP (Research Ethics Commission). The results are the main research output and, at the same time, they generate knowledge to be transmitted in the classroom.	Link

In treating variables missing in SISREHUF, the study used the highest value (input variable); the lowest value (output variable); and the mean value (link and carry-over variables).

## RESULTS

We observed heterogeneity in size and care-related characteristics of these hospitals. Mean care production (8.2% for adjusted outpatient visits and 8.0% for adjusted admissions) and technological structure (16.1%) increased over the period, despite the decrease in the number of active beds (3.7%) and non-medical staff (1.8%). There was an increase in the number of multi-professional residents (193.0%), of medical residents (21.8%) and of undergraduate students in health areas (13.9%) in the teaching dimension. Reported research projects fell by 64.7%, with no proportional fall in graduate students (increase of 1.5%) in the research dimension. We observed no significant numerical change in the number of professors with or without doctoral degrees or of doctors in most institutions (the high mean professor percentage is due to two hospitals with extreme reported values) (Table 2).

**Table 2:** Description of variables of the dynamic network DEA model applied to university hospitals. Brazil, 2010-2013.

Year	Measure	R/C Link	Care dimension (C)				T/C Link	Teaching dimension (T)				T/R Link	Research dimension (R)	
					
		Adjusted admissions (per 100)	(I) Technological structure	(I) Beds	(I) Human resources	(O) Adjusted outpatient visits (per 1,000)	Medical residents	(I) Doctors	(C) Professor without PhD	(O) Multi-professional residents	(O) Undergraduate students	Research projects	(C) Professor with PhD	(O) Graduate students
2010	Mean	132	822	295	1,708	308	159	292	83	21	1,127	184	112	256
	Maximum	597	2,355	745	4,217	2,373	792	1,084	151	68	2,327	1,868	1,002	3,362
	Minimum	3.5	31	119	501	3	8	71	18	7	197	5	5	4
	SD	157	712	162	1,051	499	151	225	38	15	484	328	175	610
2011	Mean	137	838	299	1,724	295	167	299	102	38	1,230	179	111	300
	Maximum	585	2.395	737	4,291	1.973	816	1.159	635	90	2,781	1,722	568	3,413
	Minimum	1	21	93	484	2	16	82	26	17	408	13	10	3
	SD	145	666	163	1,083	405	155	235	105	19	652	312	111	626
2012	Mean	131	916	2,856	1,662	310	177.6	287	92	50	1,213	188	111	206
	Maximum	498	2,478	734	4,557	1.842	867	1.230	487	123	3,063	1,609	575	2,246
	Minimum	0	35	96	303	0	19	19	21	7	235	6	17	1
	SD	133	748	155	1,072	442	162	243	81	31	644	298	111	405
2013	Mean	143	954	284	1,677	333	194	299	98	61	1,283	65	130	260
	Maximum	537	2,483	732	4,750	1,740	1,058	1.202	740	284	3,198	588	553	2,416
	Minimum	1	41	97	598	1	27	69	11	12	57	2	7	3
	SD	161	759	151	1,073	463	191	241	126	61	704	103	121	484

Source: Brazilian Ministry of Education Information System (SISREHUF).

DEA: data envelopment analysis; I: input variable; O: output variable; C: carry-over variable

LINK (link variables, i.e., those that are inputs for a given dimension and outputs for another dimension): Admission adjustments (R/C research input and care output); Medical Residents (T/C teaching output and care input); Research Projects (T/R teaching input and research output).

**Figure 2:**
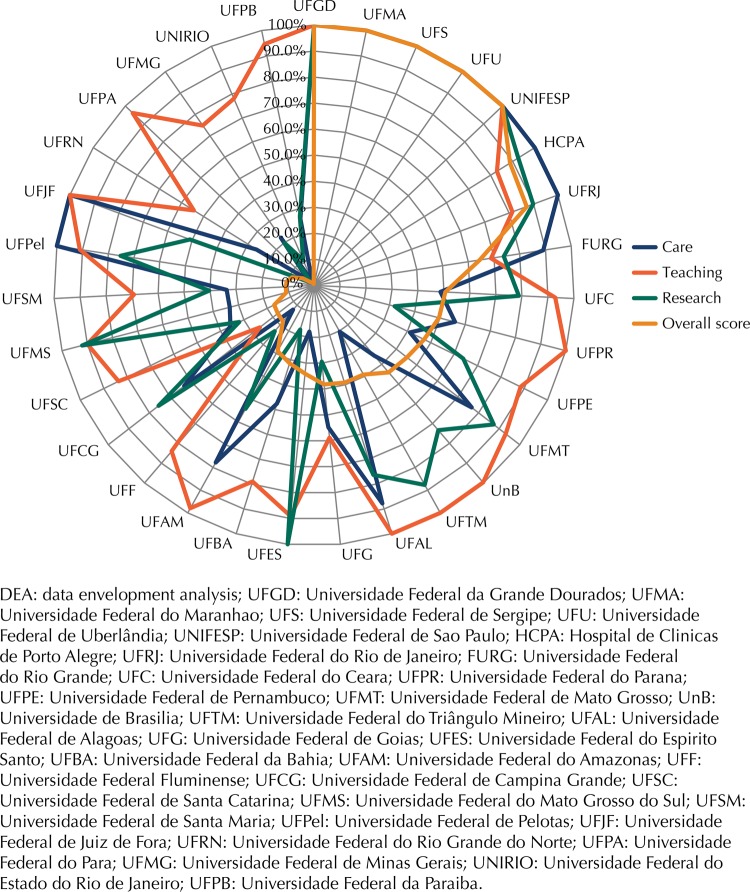
Efficiency scores of university hospitals according to the dynamic network DEA. Brazil, 2010-2013.

The overall mean efficiency score of the university hospitals in 2010, 2011, 2012, and 2013 was, respectively, 43.3%, 55.8%, 63.6%, 55.3%. Mean efficiency in all dimensions increased up to 2012, followed by a slight drop in 2013 (catch-up component). However, the frontier shift varied between the dimensions. In care, the shift fluctuated between regression in the 2010-2011 and 2012-2013 biennia and progression in the 2011-2012 biennium (mean score of 58.0%); teaching progressed in all biennia, especially 2010-2011, with the highest mean score (86.0%); the research frontier remained stationary (mean score of 61.0%) (Figure 2).

Hospitals of different sizes coexisted as benchmarks of best practice (100% score). This indicated the existence of at least two frontier regions of different scales, one with large hospitals of over 200 beds, which were efficient due to high output and complexity, and the second with smaller hospitals, with maximum efficiency measured by low resource consumption. These differences are important for the definition of peers and the choice of benchmarks of inefficient units. Five hospitals were present in the best practice frontiers (Universidade Federal do Maranhao [UFMA], Universidade Federal de Uberlandia [UFU], Universidade Federal de Sao Paulo [UNIFESP], among large hospitals; Universidade Federal de Sergipe [UFS], Universidade Federal da Grande Dourados [UFGD], among small hospitals) and six were efficient in at least two years of the studied period (Hospital de Clinicas de Porto Alegre [HCPA], Universidade Federal do Rio de Janeiro [UFRJ], among large hospitals; Fundacao Universidade do Rio Grande [FURG], Universidade Federal de Alagoas [UFAL], Universidade Federal do Amazonas [UFAM], Universidade Federal de Pelotas [UFPel], among small hospitals) (Figure 2).

**Figure 3:**
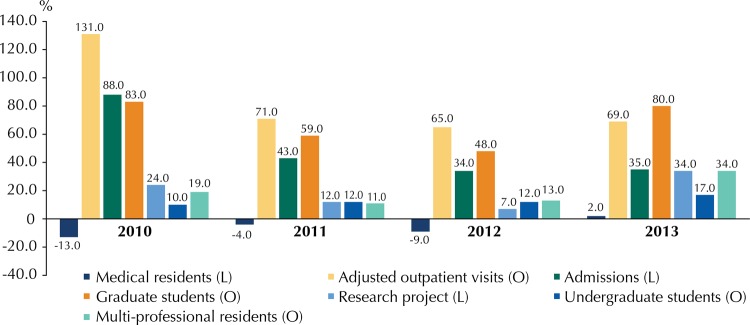
Variation need of output (O) and link (L) variables to reach best practice frontier. Brazil, 2010-2013.

The frontier projection suggested possible priorities and goals to be agreed on between managers and UHs. In 2010, at the beginning of the National Program for Restructuring Federal University Hospitals, there was a pressing need to increase output, especially in adjusted outpatient visits (131.0%) and admissions (88.0%), besides graduate students (83.0%). In 2013, the greater need was for an increase in research activities, which additionally require a significant increase in graduate students (80.0%) and care activities for outpatients (69.0%) (Figure 3).

Based on the best performance year (2012), it would be necessary to increase adjusted outpatient visits by 65.0% in care; multi-professional residence students by 13.0% and undergraduate health students by 12.0% in teaching; and graduate students by 48.0% in research for all hospitals to reach the frontier. Hospitals needing to increase output in outpatient visits or add a higher percentage of complexity in care would be Universidade Federal da Paraiba (UFPB), Universidade Federal do Estado do Rio de Janeiro (UNIRIO), Universidade Federal do Para (UFPA), Universidade Federal Fluminense (UFF), and Universidade Federal do Espirito Santo (UFES). Increase in the number of multi-professional residents would be more urgent at Universidade Federal de Campina Grande (UFCG), Universidade Federal de Minas Gerais (UFMG), UFRJ, Universidade Federal do Rio Grande do Norte (UFRN), and Universidade Federal de Santa Catarina (UFSC); increase in the number of undergraduate students at HCPA, UFCG, UFG, and UFSM, in teaching. UFAM, Universidade Federal da Bahia (UFBA), UFPA, UFPB, UFRN, and UNIRIO should have a significant increase in the number of graduate students in teaching.

Considering the number of medical residents as a link variable (care input and teaching output), the necessary values could be negative. For all hospitals to reach the frontier in 2012, there should be a mean reduction of 9.0% in the number of residents. Hospitals such as UFSC and UFCG would need to increase their numbers, but hospitals such as UFBA, UFF, Universidade Federal de Goias (UFG), UFMG, UFRN, Universidade Federal de Santa Maria (UFSM), UNIRIO, and Universidade de Brasilia (UnB) could reduce theirs without harming efficiency. As to the other link variables, adjusted admissions should increase by 34.0% and research projects by 7.0%, on average.

Table 3 presents, besides the overall score and the specific score for care, the financial budget, in millions of reais, of each UH, and the projection for the following year to reach the efficiency frontier. The approach of making projections for the best practice frontier generated financial planning guidelines based on the care output efficiency observed in each hospital.

**Table 3:** Score and budget projection of university hospitals in the care dimension. Brazil, 2010-2013.

DMU	Overall score	Care score	2010			2011			2012			2013		
			
			Value*	Projection	Dif.	Value*	Projection	Dif.	Value*	Projection	Dif.	Value*	Projection	Dif.

	%	%			%			%			%			%
UFGD	100	100	0.6	0.6	0	2.6	2.6	0	21.2	21.2	0	12.3	12.3	0
UFMA	100	100	64.8	64.8	0	80.5	80.5	0	181.9	181.9	0	140.3	140.	0
UFS	100	100	8.8	8.8	0	13.8	13.8	0	18.1	18.1	0	21.2	21.2	0
UFU	100	100	85.0	85.0	0	88.3	88.3	0	103.4	103.4	0	98.4	98.4	0
UNIFESP	100	100	135.9	135.9	0	119.5	119.5	0	176.2	176.2	0	181.9	181.9	0
HCPA	88.8	100	123.6	123.6	0	167.5	167.5	0	186.8	186.8	0	155.0	155.0	0
UFRJ	87.2	100	60.0	60.0	0	63.9	63.9	0	57.2	57.2	0	85.5	85.5	0
FURG	64.4	89.1	15.4	16.7	8.3	18.7	18.7	0	29.0	29.0	0	30.2	30.2	0
UFC	50.5	48.7	43.4	38.4	-11.5	55.6	48.0	-13.5	45.2	38.0	-15.8	62.1	52.9	-14.8
UFPR	49.6	56.0	81.9	74.3	-9.3	149.2	78.8	-47.2	104.7	104.5	-0.1	106.4	106.9	0.5
UFPE	47.2	41.1	68.6	44.7	-34.9	56.2	51.0	-9.3	48.6	61.6	26.8	96.9	66.4	-31.5
UFMT	45.2	76.6	11.7	11.7	0.0	18.8	18.8	0.0	16.8	16.8	0	13.5	13.5	0.0
UnB	44.0	35.8	49.4	29.2	-40.9	76.7	35.6	-53.6	58.6	42.8	-27.0	45.2	54.0	19.5
UFTM	39.2	20.4	46.3	42.0	-9.3	58.2	53.5	-8.2	16.2	55.9	245.8	63.5	53.1	-16.4
UFAL	39.2	87.8	21.2	21.2	0	21.2	21.2	0	22.6	22.6	0	16.1	16.1	0
UFG	38.2	54.8	38.5	44.7	16.0	63.1	44.3	-29.8	56.4	46.0	-18.3	58.8	46.0	-21.6
UFES	34.1	17.9	42.8	31.5	-26.4	48.9	40.4	-17.5	46.4	46.1	-0.6	33.3	31.3	-6.1
UFBA	31.3	47.6	40.5	38.9	-3.9	69.3	49.6	-28.5	62.7	52.1	-16.8	70.2	40.9	-41.8
UFAM	28.8	77.8	14.1	14.1	0	33.4	33.4	0	23.2	23.2	0	19.6	19.6	0
UFF	18.3	12.3	27.7	37.9	37.0	47.3	38.1	-19.6	39.6	44.2	11.7	31.1	55.0	76.6
UFCG	17.6	63.1	10.4	15.0	44.0	16.8	20.9	24.1	11.6	27.2	133.6	24.7	28.0	13.4
UFSC	17.0	35.8	30.8	40.1	30.4	59.3	45.9	-22.7	45.3	36.8	-18.9	49.4	39.0	-20.9
UFMS	11.0	33.5	28.2	30.3	7.4	52.7	31.1	-41.0	22.1	25.8	16.6	52.7	10.2	-80.7
UFSM	10.9	33.6	36.8	42.3	15.0	61.1	51.1	-16.5	74.5	66.9	-10.2	73.2	53.8	-26.5
UFPel	10.6	100	25.2	25.2	0	26.9	26.9	0	32.1	32.1	0	46.8	46.8	0
UFJF	8.9	100	11.9	11.9	0	23.2	23.2	0	27.4	27.4	0	28.2	28.2	0
UFRN	5.2	26.1	13.9	24.6	77.0	13.9	26.3	89.2	19.6	31.8	62.1	9.3	32.1	246.8
UFPA	1.8	3.2	26.6	29.0	9.0	41.4	32.5	-21.5	38.6	37.6	-2.6	30.0	31.0	3.4
UFMG	1.3	22.4	79.3	77.4	-2.4	99.7	81.1	-18.7	135.8	111.8	-17.7	128.6	120.2	-6.5
UNIRIO	1.0	1.7	32.0	28.8	-10.2	2.5	27.0	989.4	32.0	30.7	-4.3	2.5	39.3	1.478.9
UFPB	0.5	26.1	29.0	31.7	9.3	27.2	36.6	34.5	17.7	32.4	82.9	10.3	9.7	-5.7
Total	-	-	1,304.2	1,280.1	-1.9	1,677.4	1,469.6	-14.1	1,771.5	1,788.1	0.9	1,797.1	1,718.8	-4.6

Source: Brazilian Ministry of Education Information System (SISREHUF).

DMU: Decision Making Units; UFGD: Universidade Federal da Grande Dourados; UFMA: Universidade Federal do Maranhao; UFS: Universidade Federal de Sergipe; UFU: Universidade Federal de Uberlândia; UNIFESP: Universidade Federal de Sao Paulo; HCPA: Hospital de Clinicas de Porto Alegre; UFRJ: Universidade Federal do Rio de Janeiro; FURG: Universidade Federal do Rio Grande; UFC: Universidade Federal do Ceara; UFPR: Universidade Federal do Parana; UFPE: Universidade Federal de Pernambuco; UFMT: Universidade Federal de Mato Grosso; UnB: Universidade de Brasilia; UFTM: Universidade Federal do Triângulo Mineiro; UFAL: Universidade Federal de Alagoas; UFG: Universidade Federal de Goias; UFES: Universidade Federal do Espirito Santo; UFBA: Universidade Federal da Bahia; UFAM: Universidade Federal do Amazonas; UFF: Universidade Federal Fluminense; UFCG: Universidade Federal de Campina Grande; UFSC: Universidade Federal de Santa Catarina; UFMS: Universidade Federal do Mato Grosso do Sul; UFSM: Universidade Federal de Santa Maria; UFPel: Universidade Federal de Pelotas; UFJF: Universidade Federal de Juiz de Fora; UFRN: Universidade Federal do Rio Grande do Norte; UFPA: Universidade Federal do Para; UFMG: Universidade Federal de Minas Gerais; UNIRIO: Universidade Federal do Estado do Rio de Janeiro; UFPB: Universidade Federal da Paraiba.

* In millions of Brazilian reais.

The mean budget of the hospitals increased from R$42.1 to R$58.0 million over the period (mean increase of 37.8%). The largest gross increase was of UFMA (R$75.5 million) and the largest proportional increase was of UFGD (1,943.0%). Six units reported a lower budget in 2013 compared to 2010: UFAL (-24.2%), UFES (-22.2%), UFPB (-4.4%), UFRN (-33.3%), UnB (-8.5%), and UNIRIO (-92.2%). Given the fluctuation of the values reported by these units, there may be a recording mistake.

In 2013, projections for the following period suggested that seven DMUs would need a budget increase (a total of R$97.2 million), 11 would need a reduction (a total of R$175.6 million) and the others could keep it at the same level. All other parameters being the same, there would be scope for a 4.6% reduction in the total amount of funds apportioned to these hospitals in 2014, without any impact to the system’s efficiency. These guidelines would allow negotiations to increase production or redistribute resources among the units. The best performance year (2012) was the only one in which the projected funds exceeded the actual budget, i.e., the output justified the budget in a year of greater efficiency.

## DISCUSSION

The proposed model enables managers to monitor efficiency, plan goals, and establish budget guidelines over time, thus being a tool to support public policy in the field of health.

As shown in Figure 3, the projections for each year generated parameters and goals to be reached in the following period. For example, comparing the 2012 goals with the actual 2013 data, there was an increase in 20.0% in medical residents (against a reduction guideline of 9.0%). For the remaining link variables, adjusted admissions were 19.0% lower than the goal and research projects were 67.0% lower. For the remaining outputs, adjusted outpatient visits were 35.0% lower than expected; undergraduate students, 5.0% lower; graduate students, 15.0% lower. Multi-professional residents, on the other hand, were 7.0% higher than the goal. Such findings partly explain the drop in mean efficiency scores in the three dimensions in 2013.

The comparison between general hospitals, with and without teaching and research activities, not considering these dimensions, minimizes the latter’s efficiency scores. The reason is that various resources of the production process are, in practice, also geared towards non-care activities^4^. The network DEA model, on the other hand, enables comparison of the portion of resources used in each specific dimension, reducing this skew.

Since 2000, university hospitals have shown an effort in academic production, initially with classic DEA models to evaluate the efficiency of MEC units^12^. New analysis approaches have been developed for this set of hospitals, with the dimensions either treated separately or grouped in a hierarchical model or by networks^7^ ^,^ ^10^ ^,^ ^13^. Longitudinal evaluation has been used to analyze the impact of the funding reform created by the certification policy for university hospitals^9^.

The networks model was enhanced in this study by addressing the research dimension, considering only projections in the Pareto-efficient regions of the frontier and monitoring the frontier shift over the years. Such enhancement makes the model a more accurate tool for managers to analyze efficiency.

The tool proposed to monitor teaching hospitals enables managers to: a) generate efficiency scores, overall and for each dimension; b) monitor annual performance changes, overall and for each dimension; c) define and manage goals to improve performance based on the best practice frontier projection; d) program and evaluate dynamically the funding of UHs, based on efficiency. The findings have produced examples for each one of these functions. The data and projections can be analyzed separately, for each unit, or jointly, for a broader assessment of public policy.

The current set of federal university hospitals is heterogeneous in size, output capacity and operation time. Dealing with such differences required adjusting for complexity.

There was an increase in adjusted care output of approximately 8.0% in the last few years. The efficiency analysis shows that use of some resources increased simultaneously, and in a greater proportion, such as technological structure (16.1%) and funding (37.8%). On the other hand, there was a reduction in the number of beds, following a world trend in the past few years, with an increase of those in areas of greater complexity, such as intensive care units. The performance of procedures geared towards outpatients also reduced the need for beds, but there is scope to expand this activity (goal of 69.0% in 2013).

The tripled number of multi-professional residents in teaching output coincides with the policy to implement this modality over the period. In research there was a significant reduction in reported research projects, notwithstanding the quality limitations of the record.

Mean efficiency in UHs increased in all dimensions between 2010 and 2012, with a slight drop in the following year. The frontier shift, on the other hand, was heterogeneous, with higher performance and progress trend in teaching. Due to the recent implementation of correction measures in the UHs, some degree of oscillation is expected in the care frontier until the new management models are stabilized. This finding is consistent with the study of the British National Health System (NHS), which showed a contraction in the first years following its opening to the private market, later followed by an expansion of the efficiency frontier^11^.

Unlike with output-oriented goals, the definition of proposed goals considers used resources, peers in scale size, the dynamic variation of peers, and the influence of the whole group being compared. These factors enable a planning better adapted to the reality of each hospital, the definition of priorities and performance monitoring. Thus, they can serve as an additional parameter in physical and budget negotiations between unit managers and health managers, or funding agencies. The latter would be able to monitor the system’s behavior as a whole and publish sharing parameters and funding models that consider criteria related to transparency, equity and needs^17^.

The planning tool will only be valid if based on an accurate and reliable information system^6^. Inadequate records, insufficient use and lack of criticism of the system might be the main limitations of the study’s outcomes. The results and the empirical frontier vary according to the set of variables used. Hence the importance of having various actors involved in evaluating the model’s construction, so that each element of the model is consensually approved for the several hospital profiles to be compared.

## Footnotes

a Ministério da Saúde. Portaria nº 3.390, de 30 de dezembro de 2013. Institui a Política Nacional de Atenção Hospitalar (PNHOSP) no âmbito do Sistema Único de Saúde (SUS), estabelecendo-se as diretrizes para a organização do componente hospitalar da Rede de Atenção à Saúde (RAS). *Diario Oficial Uniao.* 31 dez 2013; Seção 1:54-6.

b Ministério da Saúde. Portaria interministerial nº 2.400, de 2 de outubro de 2007. Estabelece os requisitos para certificação de unidades hospitalares como Hospitais de Ensino. *Diario Oficial Uniao*. 3 out 2007; Seção 1:102-3.

c Brasil. Decreto nº 7.082, de 27 de janeiro de 2010. Institui o Programa Nacional de Reestruturação dos Hospitais Universitários Federais-REHUF, dispõe sobre o financiamento compartilhado dos hospitais universitários federais entre as áreas da educação e da saúde e disciplina o regime da pactuação global com esses hospitais. *Diario Oficial Uniao*. 27 jan 2010; Edição extra; Seção 1:1-2.

d Brasil. Lei nº 12.550, de 15 de dezembro de 2011. Autoriza o Poder Executivo a criar a empresa pública denominada Empresa Brasileira de Serviços Hospitalares - EBSERH; acrescenta dispositivos ao Decreto-Lei nº 2.848, de 7 de dezembro de 1940 - Código Penal; e dá outras providências. *Diario Oficial Uniao*. 16 dez 2011; Seção 1:2-3.
